# Management of uncomplicated malaria in children under 13 years of age at a district hospital in senegal: from official guidelines to usual practices

**DOI:** 10.1186/1475-2875-10-285

**Published:** 2011-09-29

**Authors:** Sophie Sarrassat, Richard Lalou, Moustapha Cissé, Jean-Yves Le Hesran

**Affiliations:** 1 IRD, UMR 216 « Santé de la mère et de l'enfant en milieu tropical », Université Pairs Descartes, Faculté de Pharmacie, Paris, France; 2 IRD, UMR 151 « Laboratoire Population Environnement Développement », Marseille, France; 3 Programme National de Lutte contre le Paludisme, Bureau Prévention et Partenariat, Dakar, Sénégal

## Abstract

**Background:**

To be effective, national malaria guidelines must be properly followed. This study evaluated nurses' practices in the management of uncomplicated malaria cases at a District Hospital. Its objective was to identify the reasons for discrepancies between official guidelines and usual practices.

**Methods:**

This study took place at Oussouye hospital, south-western Senegal. Blood smears were available for biological diagnosis in patients aged more than five years while the Integrated Management of Childhood Illness recommended treating fevers presumptively in children under five. First line anti-malarial was Amodiaquine plus sulphadoxine-pyrimethamine (AQ+SP) bi-therapy. Hospital records of children under 13 years of age seen between 2004 and 2005 were reviewed.

**Results:**

Among children treated with anti-malarials, 74% (2, 063/2, 789) received AQ+SP. However, only 22% (406/1, 879) of febrile children and 19% (429/2, 198) of children diagnosed with malaria got a blood smear. Moreover, an anti-malarial was prescribed for 80% (377/474) of children with a negative blood smear.

**Conclusions:**

The transition from chloroquine to AQ+SP was well followed. Nonetheless, blood smear use was very low and many over-prescriptions were reported. Reasons for discrepancies between guidelines and practices can be classified in three main categories: ambiguous guidelines, health system's dysfunctions and nurses' own considerations. Aside from the strengthening of the public health system, in order to guarantee practices complying with guidelines, training content should be more adapted to nurses' own considerations.

## Background

In the past, while first and second line recommended anti-malarials were chloroquine (CQ), sulphadoxine-pyrimethamine (SP) or amodiaquine (AQ) mono-therapy, blood smears were found to be infrequently used despite being available, and non-recommended prescriptions were frequent. In Zambia in 1997, a blood smear was requested for only 46% of patients with fever or previous fever, and among those with a negative result, 35% were treated with an anti-malarial [1]. In Kenya in 2002 [2] and in Tanzania in 2004 [3], this percentage was 79% and 48% respectively. Moreover, Barat *et al *[1] and Zurovac *et al *[2] reported 11% and 43% respectively of patients without any history of fever treated with an anti-malarial. As far as the choice of prescribed anti-malarial is concerned, three studies showed prescriptions not complying with guidelines. In Benin in 1999 [4], in Tanzania in 2003 [5] and in Kenya in 2001 [6], quinine (QN), although restricted to severe malaria, was prescribed for 33%, 19% and 12% respectively of children under five treated with an anti-malarial. Moreover, in Kenya, 15% of children received a combination of CQ, AQ or SP.

Only a few studies, however, researched the reasons for these non-recommended practices [1,7,8]. This study, therefore, evaluated nurses' practices in the management of uncomplicated malaria cases at a District Hospital in Senegal. Its objective was to identify the reasons for discrepancies between official guidelines and usual practices.

## Methods

### Study site

This study took place from July to September 2005 at the District Hospital of Oussouye, south-western Senegal. In this area, malaria is meso-endemic with a seasonal recrudescence of cases during the rainy season, from June to October. The hospital was managed by a physician and was the referral centre for twelve dispensaries within the district. Two out-patient consultation rooms managed by nurses were available, one for children and one for adults. Consultations with a physician were also offered in a third room. The hospital had a laboratory facility managed by a skilled technician and equipped for blood smears. These services were open from Monday to Friday, 8:30 to 13:00.

At the time of this study and since 2003, national malaria guidelines recommended AQ+SP bi-therapy for treating uncomplicated malaria [9]. In addition, in children under five, the Integrated Management of Childhood Illness (IMCI) advocated presumptive treatments of fevers or previous fevers [10]. In patients over five, healthcare providers should make a differential diagnosis of fevers. Where blood smears were available, this biological diagnostic tool had to be used.

### Collection and analysis of data from Hospital records

A total of 5, 441 consultations involving children younger than 13 years old diagnosed with malaria or treated with an anti-malarial were retrospectively extracted from 2004 and 2005 hospital records. For each of these consultations, following data were recorded: out-patient consultation room, date, age, sex, axillary temperature, blood smear result if requested, diagnosis and prescribed anti-malarial. History of previous fever was not consigned in consultation records.

Nine point five percent (517/5, 441) of extracted consultations were excluded from the analysis: 235 consultations involving infants less than two months old, the IMCI recommending to treat fevers with antibiotics in this age group, and 282 consultations with missing data (age, diagnosis or treatment). A total of 4, 924 consultations were, therefore, analysed: 2, 371 in 2004 and 2, 553 in 2005.

Blood smear use was determined according to the presence of fever (axillary temperature ≥ 37.5°C) and diagnosis made by nurses, malaria or other illness. Two aspects of anti-malarial prescription practices were analysed: The type of anti-malarials prescribed and the type of patients treated, febrile or not, with a positive or negative blood smear, and diagnosed with malaria or another illness. These analyses were performed in two age groups: under-five and from five to 13 years old. Statistical analyses were performed using STATA10. Pearson's chi square tests were used to compare two percentages (α = 0.05).

## Results

**Figure 1:**
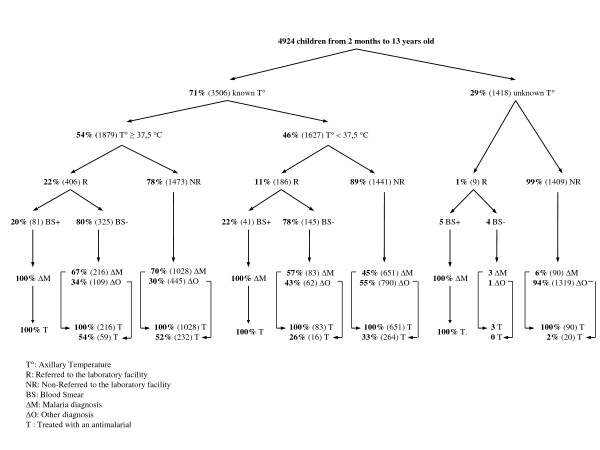
**Children's healthcare according to the chronological order of events during consultations**. T°: Axillary temperature. R: Referred to the laboratory facility. NR: Non-referred to the laboratory facility. BS: Blood smear. ΔM: Malaria diagnosis. ΔO: Other diagnosis. T: Treated with an anti-malarial.

Figure 1 shows children's healthcare according to the chronological order of events during consultations: temperature measurement, blood smear request, determined diagnosis and anti-malarial prescription. 57% (2, 814/4, 924) of children were under five. Eighty two percent (4, 041/4, 924) of children were seen in the paediatric consultation room, 17% (853/4, 924) in the adult consultation room and 1% (30/4, 924) in the physician's room. Nurses measured the temperature of 71% (3, 506/4, 924) of children. Overall 54% (1, 879/3, 506) were febrile, 57% (1, 144/2, 009) of children under five and 49% (735/1, 497) of children from five to 13 years old (p < 0.0001).

### Blood smears use

Only 22% (406/1, 879) of febrile children were referred to the laboratory facility for a blood smear. Their mean axillary temperature was 38.3 ± 0.7°C (n = 404) versus 38.2 ± 0.8°C (n = 1, 473) in febrile but non-referred children. Moreover, only 19% (429/2, 198) of children diagnosed with malaria got a blood smear. Among them, only 30% (127/429) had a positive result.

By age, the use of blood smears was similar. Twenty three percent (262/1, 144) of febrile children under five and 20% (144/735) of febrile children from five to 13 years old were referred for a blood smear (p = 0.09). And, when they were diagnosed with malaria, 22% (224/1, 038) and 18% (205/1, 160) respectively were referred for a blood smear (p = 0.02).

Nurses also requested a blood smear in 11% (186/1, 627) of non-febrile children and 6% (172/2, 726) of children diagnosed with a non-malarial illness.

### Type of anti-malarials prescribed

**Figure 2:**
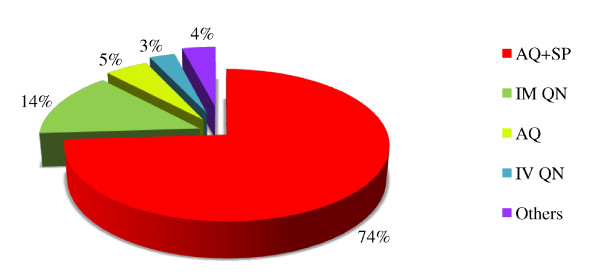
**Type of anti-malarials prescribed**. Red quarter represents the proportion of children treated with AQ+SP. Dark green quarter represents the proportion of children treated with IM QN. Pale green quarter represents the proportion of children treated with AQ. Blue quarter represents the proportion of children treated with IV QN. Purple quarter represents the proportion of children treated with other anti-malarials. Other include AS+AQ: 1.7% (48/2789); AS: 1.1% (30/2789); CQ: 0.8% (22/2789); AS+SP: 0.4% (12/2789).

The transition to AQ+SP was well followed, 74% (2, 063/2, 789) of children treated with an anti-malarial received this bi-therapy (Figure 2). From 2004 to 2005, its use increased from 64% (926/1, 438) to 84% (1, 137/1, 351) (p < 0.0001). Mono-therapy prescriptions were minor, 5.0% (149/2789) for AQ, 1.1% (30/2789) for Artesunate (AS) and 0.8% (22/2789) for CQ. Other anti-malarial treatments were very rarely prescribed, 1.7% (48/2789) for AS+AQ and 0.4% (12/2789) for AS+SP. Regarding the use of QN, 3% (81/2789) of treated children received IV injection and 14% (379/2789) IM injection.

### Type of patients treated with an anti-malarial

According to the presence or not of fever, nurses prescribed an anti-malarial to 86% (1, 616/1, 879) of febrile children and 65% (1, 055/1, 627) of non-febrile children (table 1). Among non-febrile children who received an anti-malarial, 73% (775/1, 055) were diagnosed with malaria. According to the results of blood smear, 100% (127/127) of children with a positive result and 80% (377/474) of children with a negative result were treated with an anti-malarial. Among those with a negative blood smear, 74% (275/374) were febrile and 80% (302/377) were diagnosed with malaria.

**Table 1:** Types of patients treated

	No. children	% treated (n)
**Febrile**	1 879	86.0 (1616)
**Non febrile**	1 627	64.8 (1055)
**Missing temperature**	1 418	8.3 (118)

**Positive blood smears**	127	100.0 (127)
**Negative blood smears**	474	79.5 (377)

**Malaria diagnosis**	2 198	100.0 (2198)
**Other diagnosis**	2 726	21.7 (591)

**Total of consulted children**	4 924	56.6 (2789)

Lastly, given the diagnosis recorded by nurses, 100% (2, 198/2, 198) of children diagnosed suffering from malaria and 22% (591/2, 726) of children diagnosed suffering from a non-malarial illness received an anti-malarial. Among those treated while diagnosed with a non-malaria illness, 51% (291/571) were febrile. The main diagnosis recorded were respiratory infections, for 76% (452/591), or gastro-intestinal infections, for 21% (123/591).

In children under five and in those from five to 13 years old respectively, the percentages of treated children were 60% (516/865) and 71% (539/762) in the absence of fever (p < 0.0001); 77% (235/305) and 84% (142/169) when blood smears were negative (p = 0.07); 27% (472/1, 776) and 13% (119/950) when a non-malarial illness was diagnosed (p < 0.0001). Overall, 54% (1, 510/2, 814) of children under five and 61% (1, 279/2, 110) of those aged from five to 13 received an anti-malarial (p < 0.0001).

## Discussion

In Senegal, artemisinin-based combination therapy (ACT) and rapid diagnostic tests (RDT) have been implemented in 2006 after a transitory stage recommended AQ+SP bi-therapy [11]. This study took place at a District Hospital during this transitory stage and allowed evaluation of the context of uncomplicated malaria cases management into which ACT and RDT were later introduced. It identified discrepancies between official guidelines and nurses' usual practices and tried to explain their determinants.

### Compliance with the transition to AQ+SP bi-therapy

The transition to AQ+SP was well followed at Oussouye Hospital. This result can be attributed to the favourable context of prescription in place in a District Hospital. Indeed, nurses were trained and regularly supervised by the hospital physician. Moreover, stocks of CQ were removed and those of AQ+SP never experienced shortage.

These good practices contrast with those reported in 2004, in Thies, north-western Senegal, where nurses prescribed AQ or SP mono-therapy for 15% to 78% of presumptive malaria cases [12]. Similarly, in Zambia in 2004 and in Kenya in 2006, only 13% and 34% of treated children received artemether/lumefantrine (A/L), the first line anti-malarial [13,14]. Even if these three studies reported prescription practices while the new therapy has been recommended for a few months, in Zambia, two years later, only 47% of treated children received A/L [15]. In contrast to this study, health facilities studied were dispensaries. To explain their results, authors reported health system's dysfunctions such as the availability of previous recommended anti-malarials, shortages of new anti-malarials and a lack of medical staff's training and supervision. While "cascade" training had been planned at the time of new guidelines deployment, in Senegal [12] and Kenya [8], it was delayed at the lowest level of the health pyramid by reason of lack of Global Fund subventions. The facility's location on the health pyramid seems therefore to be a major component. The higher the facility is located up the pyramid, the more it benefits from a favourable context for good healthcare practices.

In addition, to justify non-recommended prescriptions, nurses from Thies [12] and Kenya [8] explained they deviated from guidelines in response to certain practical or clinical situations. These situations encompassed the risk of an excessive dose in case of repeated prescriptions of SP, the paediatric forms' convenience, patients' demands or adjusted prescriptions in case of high fevers or adverse events of AQ.

In Oussouye, informal interviews with nurses showed similar concerns and the extent to which guidelines describe standard clinical cases while nurses are confronted with various individual situations, which can lead to non-recommended prescriptions. In the paediatric consultation room, nurses preferred to prescribe AQ alone when SP had been taken in the previous month to adhere to the one-month minimum interval recommended by the Intermittent Preventive Therapy during pregnancy. When patients had started to self-medicate with CQ, AQ or AS, nurses could carry on with this treatment, conscious of some households' low incomes. In young children, they could choose to prescribe AS suppositories because of their convenience. In the adult consultation room, in cases of high fever, one particular nurse preferred to prescribe AS because of its effectiveness. Lastly, all nurses explained that pressure from parents can sometimes affect their prescribing. Parents wanted IM QN because it was perceived as being more effective or AQ drinkable suspension to store at home for treating fevers. Others refused AQ because of its adverse effects such as asthenia, stomach aches, nausea or itching.

To conclude, it seems there are two kinds of potential impediments to the compliance with guidelines, first linked to the running of the health facility or its level in the health pyramid, and second linked to medical staff's handling of situations that they encounter daily with their patients.

### Low use of blood smears and over-prescriptions

At Oussouye Hospital, compliance with the transition to AQ+SP has to be balanced. Indeed, while a laboratory facility was available, a very small proportion of patients got a blood smear. In addition, three types of over-prescription were identified. Sixty five percent (1, 055/1, 627) of non-febrile children were treated. However, as no history of previous fever was consigned in consultation records, these over-prescriptions are probably over-estimated. Secondly, 22% (591/2, 726) of children diagnosed with a non-malaria illness were treated. These over-prescriptions explain why 57% (2, 789/4, 924) of children received an anti-malarial, while only 45% (2, 198/4, 924) were diagnosed with malaria. Lastly, and more surprisingly, 80% (377/474) of children received an anti-malarial despite their negative blood smear. These over-prescriptions explain why 84% (504/601) of children referred to the laboratory facility for a blood smear received an anti-malarial, while the positive blood smear percentage was only 21% (127/601).

In a context of RDT deployment, similar results were reported. In Zambia in 2006, about one year after their introduction, only 23% of patients with fever or previous fever were tested. Moreover, 36% of patients with a negative RDT were treated with an anti-malarial [16]. In Tanzania in 2005, this proportion rose to 54% [17]. Despite their many advantages, the use of RDT could therefore encounter similar problems to those identified with blood smears.

Considering observations made at Oussouye Hospital and informal interviews with its medical staff, three spheres of influence seem to lead to these discrepancies in guidelines. Firstly, an ambiguous guideline enacted by the hospital physician, recommending to treat fevers with a negative blood smear as "*a precaution*" if no other illness was diagnosed, probably affected nurses' prescribing. This type of ambiguous guideline is officially found in other sub-Saharan countries [7,18]. In Zambia, Hamer *et al *[16] also pointed out the ambiguity of the guideline in the case of negative results. However, from the clinicians' point of view, it is justified by the risk of severe malaria in cases of false negative result.

Furthermore, the multiplicity of guidelines, with IMCI, could also contribute to over-prescriptions. Indeed, this strategy recommends presumptive treatments of fevers in children under five to reduce malaria mortality. Nurses could, therefore, have applied this principle to older children, considering that there is no age limit for the risk of severe malaria.

Alongside guidelines, dysfunctions at the laboratory facility also resulted in practices not complying with guidelines. Both nurses and laboratory technician pointed out the high workload required by blood smears, especially results reading time, and technical or material problems such as lack of supplies, microscope failures or power cuts. As a result, nurses confessed they were most of the time discouraged to request blood smears. Moreover, the laboratory technician explained that he was sometimes able to read blood smears only after the end of consultations leading nurses to prescribe without knowledge of their negative results. These dysfunctions have been reported in other sub-Saharan countries [1,7,19]. At two Tanzanian regional hospitals, they led to both a low use of blood smears and a delay in reading 56% of referred patients' slides [7].

While RDT do not require either microscope or electricity and hence avoid material problems, their usage still requires time, in particular at least 15 minutes before the results can be read. At dispensaries where the mean consultation time is often very short, in particular five minutes or less during the rainy season in Kenya or in Tanzania [2,20], it seems probable that nurses, who have to perform these tests, take shortcuts. In Senegal, some nurses considered the delay to obtain results as being still too long [21].

Lastly, at Oussouye Hospital, nurses' own considerations seem to have played an important role in explaining both the low blood smear use and over-prescriptions. To explain low blood smear use, nurses blamed a lack of healthcare's organisation. The long period of time to obtain results led to an accumulation of patients at the laboratory and consequently delayed the end of consultations. Patients returning from laboratory also disrupted the clinic as they sometimes pushed in front of other waiting patients creating confusion and dissatisfaction. In addition, due to restricted laboratory opening times, nurses were not able to refer patients for a blood smear after 1 pm. In Zambia, Barat *et al *[1] also reported a very long consultation time, on average 57 ± 31 minutes, when blood smears were requested. Moreover, authors emphasized the absence of full-time laboratory facilities.

Another concern of Oussouye nurses was that patients refused to have a blood smear due to its cost (300 CFA or 0.46 Euro) and time involved. These refusals must be interpreted thoroughly. Indeed, patients build their own diagnosis and therapeutic approach through what they learn from different sources of information, in particular practices observed during consultations [22]. Therefore, patients probably noticed that they had been given anti-malarials whether or not they had been referred and, worse, whatever the result of their blood smear. These practices probably did not convince them of the usefulness of this exam.

To justify over-prescriptions, nurses' own considerations encompassed their lack of trust in the reliability of negative blood smears due to laboratory dysfunctions and their perceptions of malaria. Indeed, beyond standard symptoms of malaria, all nurses described atypical malaria cases, such as "*non-febrile malaria*" suspected in patients with headaches, chills, asthenia or vomiting, and "*hidden malaria*" in which the blood smear is negative because parasites are not yet in the blood. Furthermore, nurses mentioned once again some situations encountered with their patients, such as parents' demands for an anti-malarial or previous self-medication with an anti-malarial or an antipyretic which can explain the absence of fever or the negativity of blood smear and, therefore, do not exclude an anti-malarial prescription. In Tanzania, Chandler *et al *[7] interviewed nurses who shared some of these considerations, such as the doubt about the reliability of negative blood smears, the notion of false negative results because of parasites sequestration and patients demanding an anti-malarial. Although blood smears are reliable when performed by a skilled technician, their sensitivity in routine practice can be mediocre [2,3,23]. Besides technical or material problems, the lack of qualified laboratory staff is another frequent reason for unreliable blood smear results [24]. In such a context, the lack of confidence expressed by nurses cannot be blamed.

Although RDT are considered reliable, nurses could also doubt their negative results. In Tanzania, within routine practice, their sensitivity was evaluated between 19% and 86% [25]. In this country and in Sudan [26], three types of error were frequently observed: inadequate capillary blood sample volume, incorrect waiting time to read results and low intensity positive results read as negative. Moreover, inadequate storage conditions, such as temperatures above 35°C or humidity, frequent in sub-Saharan Africa, can also affect the quality of RDT [27].

To summarise, over-prescriptions seem to be explained by practical, technical or socio-cultural considerations more than by clinical considerations. Nonetheless, as an underlying concern, interviews with Oussouye nurses also revealed their fear of severe malaria in non-treated patients. In Tanzania, Chandler *et al *[7] revealed the same dogma: malaria is a frequent and fatal illness that nurses have to treat as a priority. This fear can be explained by personal experiences, campaigns against malaria highlighting the lethality of this disease, but also by the training of medical staff which put a slant on the risk of severe malaria. In order to rationalize prescriptions, it therefore seems important to consider this fear and appease it.

## Conclusion

This study showed how difficult it is to integrate guidelines into nurses' daily practices. Reasons for discrepancies between official guidelines and usual practices can be classified in three main categories: the ambiguity and multiplicity of guidelines, health system dysfunctions and nurses' own considerations.

One objective of the "Roll Back Malaria" partnership for 2015 is to treat correctly 80% of patients with malaria. To achieve this objective, the WHO has advocated, since 2001, the use of ACT for treating uncomplicated malaria [28] and, since 2006, the biological confirmation of malaria cases by RDT [29]. However, in practice, impacts of these diagnostic and therapeutic interventions depend, in part, on whether the official guidelines are followed by medical staff. Therefore a favourable context for their deployment must be created. In particular, a quality control system for biological diagnosis seems essential to gain the trust of nurses. Moreover, aside from the strengthening of the public health system, there is an important need to further match both training content and tools provided to medical staff, such as algorithm, to their own considerations.

## Competing interests

The authors declare that they have no competing interests.

## Authors' contributions

SS designed the study, collected data, carried out the analysis, interpretation of data and prepared the manuscript. RL and MC contributed in the conception of the study and collection of data. JYLH contributed in the analysis and interpretation of data. All authors have participated in drafting and revising the paper and approved the manuscript.
